# Mixed gaits in small avian terrestrial locomotion

**DOI:** 10.1038/srep13636

**Published:** 2015-09-03

**Authors:** Emanuel Andrada, Daniel Haase, Yefta Sutedja, John A. Nyakatura, Brandon M. Kilbourne, Joachim Denzler, Martin S. Fischer, Reinhard Blickhan

**Affiliations:** 1 Science of Motion, Friedrich-Schiller University of Jena, Germany; 2 Computer Vision Group, Friedrich-Schiller University of Jena, Germany; 3 Institut für Spezielle Zoologie und Evolutionsbiologie mit Phyletischem Museum, Friedrich-Schiller University of Jena, Germany; 4 AG Morphologie und Formengeschichte, Bild Wissen Gestaltung: ein interdisziplinäres Labor, Institut für Biologie, Humboldt University Berlin, Germany; 5 College for Life Sciences, Wissenschaftskolleg zu Berlin, Berlin, Germany

## Abstract

Scientists have historically categorized gaits discretely (e.g. regular gaits such as walking, running). However, previous results suggest that animals such as birds might mix or regularly or stochastically switch between gaits while maintaining a steady locomotor speed. Here, we combined a novel and completely automated large-scale study (over one million frames) on motions of the center of mass in several bird species (quail, oystercatcher, northern lapwing, pigeon, and avocet) with numerical simulations. The birds studied do not strictly prefer walking mechanics at lower speeds or running mechanics at higher speeds. Moreover, our results clearly display that the birds in our study employ mixed gaits (such as one step walking followed by one step using running mechanics) more often than walking and, surprisingly, maybe as often as grounded running. Using a bio-inspired model based on parameters obtained from real quails, we found two types of stable mixed gaits. In the first, both legs exhibit different gait mechanics, whereas in the second, legs gradually alternate from one gait mechanics into the other. Interestingly, mixed gaits parameters mostly overlap those of grounded running. Thus, perturbations or changes in the state induce a switch from grounded running to mixed gaits or *vice versa*.

When researchers analyze legged locomotion, they usually categorize discretely regular gaits (i.e. walking or running) based on the relative contact time of limbs (limb phase and duty factor^1^) or fluctuations of the mechanical energy at the center of mass (CoM)^2^,^3^. The latter categorization is better suited to discriminate between walking and running (see^4^ for a deeper discussion on this topic) and served as a fruitful basis for the development of relative simple models like the inverted pendulum for walking^3^ or the spring-mass model for running^5^. These relatively simple models for walking and running have greatly widened our understanding of global principles of locomotion; however, they represent only discrete idealized paradigms. For human locomotion, such discrete changes may apply, but not necessarily for small animals, as gait transitions have often been shown to be more gradual in smaller species (e.g.^4^,^6^,^7^,^8^). In particular, smooth gait changes were described for small bird terrestrial locomotion (e.g.^6^,^7^,^8^,^9^,^10^,^11^). Contrarily, the existence of hybrid gaits (both legs exhibit a combination of two regular gaits in one stride, e.g. “pendular run”^12^) or mixed gaits (legs display different gait dynamics, i.e. one step with walking mechanics is followed by one step with running mechanics or *vice versa* at a relatively constant speed) during bipedal locomotion has received little attention. Scientists usually focus on the investigation of a regular gait and expect that deviations are due to unsteady locomotion. For example, most of the theoretical work on bipedal locomotion is focused on one-cycle periodic gaits, and likely avoid unintentionally ‘limping’ or ‘hybrid gaits’ (e.g.^5^,^13^,^14^,^15^).

The discussion about hybrid gaits emerged after numerical energetic optimizations of another simple model (point-mass and telescopic leg) predicted such a gait during locomotion at intermediate speeds utilizing long step-lengths, which was termed a ‘pendular run’^12^. More recently, Usherwood^16^ found hints of pendular running in the terrestrial locomotion of pheasants and guineafowl by analyzing their CoM motions derived from forceplate measurements. Given that the inverted pendulum^2^ is almost 40 years old and that the spring-mass model^5^ is almost 30 years old, it seems fair to wonder to what degree the walking and running paradigms influenced and still influence the selection and analysis of strides in animal locomotion? This question is of importance not only in regard of avian terrestrial locomotion, but also to terrestrial locomotion in other tetrapod groups.

It is generally accepted that birds walk at the lowest speeds, however, exceptions such as sparrows exist (see also text below). At moderate speeds, small birds usually prefer a running gait without aerial phases, which was termed grounded running^15^,^17^,^18^. Theoretically during grounded running, potential (E_p_) and kinetic (E_k_) energy of the CoM change nearly in phase (bouncing mechanics), and, consequently, a high congruity occurs between both energy patterns (%congruity as a measure for CoM energy pattern see^19^). In contrast, the vaulting motion of the CoM observed in walking produces E_P_ and E_K_ patterns that fluctuate out-of-phase (low congruity). However, zero congruity is not expected for compliant walking^13^. In small birds, the shift between vaulting and bouncing mechanics is said to be gradual, rarely approaching the ideal scenarios (e.g.,^7^,^10^). If this is the case, as speed increases, one can expect that %congruity values will cross a value of 50, which is usually defined as a boundary between walking-like and running-like gaits (e.g.^7^,^19^,^20^). In this ‘transition zone’, small fluctuations, as birds experience on a treadmill or face in irregular natural environments, might produce %congruity shifts to either walking or running mechanics. Two effects can be expected: i) in some trials running occurs at a slower speed than walking in another trial and/or ii) mixed gaits emerge.

In the present paper, we combined a large-scale study (over one million frames) on the fluctuations of the CoM’s mechanical energy in several bird species (quail, oystercatcher, northern lapwing, pigeon, and avocet) during treadmill locomotion, which was performed in a completely automated way, with numerical simulations, in order to address two main points.

First, we examined whether the transition between vaulting (i.e. walking) and bouncing (i.e. running) mechanics correlates with speed, without human influence introduced via manual digitizing and subjective choice of strides for analysis. Even though globally a trend towards increasing %congruity with higher speeds was observed in recent investigations, at lower speeds, some birds, such as lapwings^7^ and tinamous^10^, do not always seem to prefer walking mechanics. In contrast, quail^18^ and the much larger ostrich^17^ seem to rely on CoM’s mechanics indicative of walking during slow locomotion.

Second, we asked whether mixed gaits exist at the boundary between walking and grounded running. Within a range of relative speeds (Froude numbers of approximately 0.5 ~ 0.6), it is frequently observed that both walking and grounded running gaits occur in birds^7^,^17^,^18^. In addition, it was found that virtual leg stiffness (computed from the ground reaction forces and the length change between the CoM and foot contact point) remain at values close to 6 during quail walking, grounded running and running when normalized by body mass and leg-length^18^. By combining experimental data on quail and simulations, Andrada and colleagues^21^ showed that pronograde locomotion maximizes the advantages of grounded running. Moreover, they found that in order to obtain walking gaits, the effective leg, defined as the length between the hip and foot contact point, must be less stiff but more damped than a leg tuned for running. Thus, they speculated that in the quail there must occur a shift to an increasing role of elastic tissues during running as also observed in running turkeys and running humans^22^,^23^. It is therefore conceivable that during the transition between walking and grounded running, model related leg parameters such as leg stiffness or leg damping should be suitable for both gaits, thus facilitating a potential emergence of mixed gaits.

To address these questions, we analyzed X-ray recordings on a large quantitative scale using the algorithms developed by Haase and colleagues^24^. The methodology permits an automatic tracking of the CoM. In addition, foot contacts and aerial phases were also detected through automation. Then we determined for each step the percentage of congruity to differentiate vaulting from bouncing mechanics.

Finally, to test whether mixed gaits are part of the repertoire of the dynamics of pronograde bipedal locomotion, and not just a treadmill artifact, we explored numerically, based on parameters derived from quail (cf.^21^), the existence of periodic mixed gaits within the overlap zone of walking, grounded running and running by using a simple model called Pronograde Virtual Pivot Point (PVPP;^21^). In simulations both legs have same basic effective leg parameters such as leg length (*l*_0_), leg stiffness (*k*), and leg damping (*c*).

## Results

### Congruity of CoM mechanics

In general no correlation between %congruity and speed was observed. However, some differences among species can be established based on the scatter plots.

**Figure 1:**
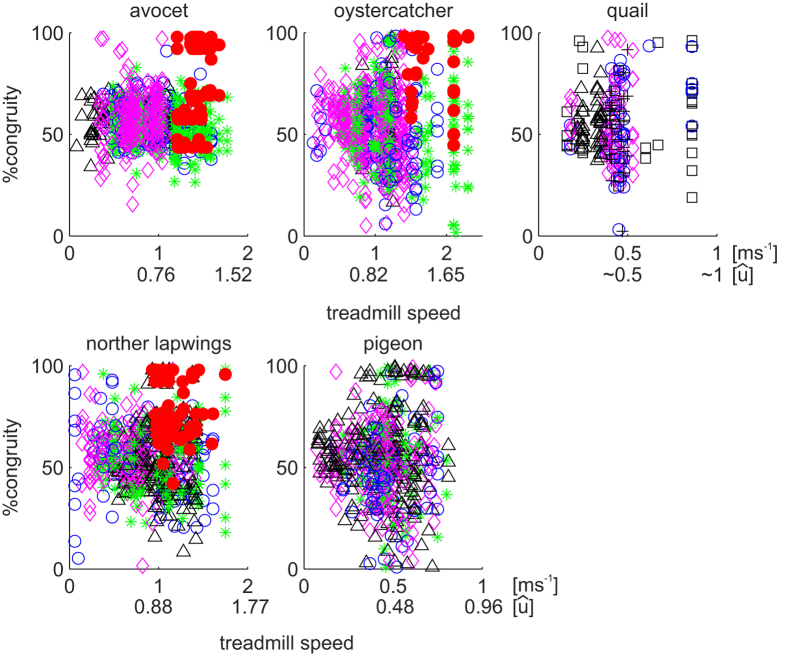
%Congruity of CoM mechanics related to treadmill and dimensionless speed (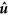). %congruity values lower up to 50 are often related to vaulting mechanics, while those larger than 50 are interpreted as bouncing mechanics. Each point represent a step. Red points indicates that aerial phases occurred.

**Figure 2:**
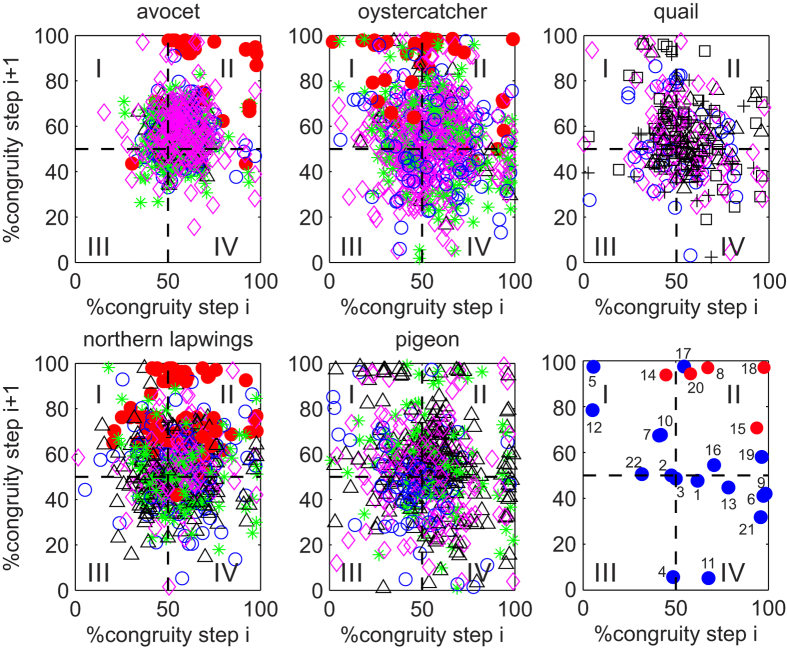
Values of %Congruity in step i vs. step i + 1. Quadrant I and IV depict mixed gaits. In I %congruity shifts from walking values in step i to values representing running mechanics in step i + 1. The inverse occurs in IV. II and III portray regular gaits of running and walking mechanics, respectively. Red points indicates that aerial phases occurred. For individual/marker type correspondence see Table 1. Bottom right shows a single trial from individual “3970(7)” comprised of 23 steps at 2.1 m s^−1^. Note that it combines mixed gaits, grounded running, running (red), and walking. Arabic numbers indicate step sequence.

We recorded trials from avocets that met the criteria for analysis at speed between 0.14 ms^−1^ and 1.77 ms^−1^. Despite this speed range, avocets seem to prefer the transition zone of % congruity values ranging between 40% and 70% (Fig. 1). No aerial phases were observed at speeds below 1.2 ms^−1^. At speeds above 1.2 ms^−1^, only two individuals ran without aerial phases, but still exhibited in 29% of steps %congruity values lower than 50. In this speed interval, 3 individuals ran with aerial phases in just 16% of the steps. When generally comparing one step with the subsequent step (Table 1 and Fig. 2), ~52% of these pairs of steps exhibited solely running mechanics in both steps, ~8% solely walking mechanics walking in both steps, and ~40% mixed walking and running mechanics between steps. Most of the aerial phases where observed in the area of two consecutive running steps. Individual differences are significant due to one individual which used relatively more walking and less bouncing mechanics during trials (Table 1). The variation observed among the other three individuals is random.

Northern lapwings displayed a pattern similar to avocets, but with slightly enhanced dispersion of the data. They moved at speeds ranging from 0.06 ms^−1^ to 1.75 ms^−1^. Northern lapwings did not incorporate aerial phases at speeds lower than 1.0 ms^−1^. At speeds above 1 ms^−1^ they used aerial phases in 23% of the steps, but in 39% of the steps %congruity was still lower than 50 (Fig. 1). When comparing consecutive steps in general, ~40% of them exhibited running mechanics in both steps, ~44% a mixture of walking and running, and ~16% walking in both steps (Fig. 2). As observed for the avocets, individual differences are significant due to one individual (B67), which used relatively more frequently walking and less bouncing mechanics (Table 1). The variation observed among the other three individuals is due to chance.

Oystercatchers and pigeons displayed a similar pattern in their scatter plots, though in different speeds ranges (oystercatchers: 0.13 ms^−1^ to 2.3 ms^−1^; pigeons: 0.07 ms^−1^ to .81 ms^−1^). Both species exhibited intermediate %congruity values (between 40 and 70) at lower speeds, followed by a greater dispersion at intermediate and high speeds. We did not record aerial phase running in oystercatchers at speeds below 1.4 ms^−1^. At speeds ranging between 1 ms^−1^ and 1.4 ms^−1^, oystercatcher exhibited in 31% of the steps %congruity values lower than 50. At speeds higher than 1.4 ms^−1^, they used aerial phases in 23% of the steps, and vaulting mechanics in 40% steps. When comparing one step with the subsequent one, 32% of them exhibited running mechanics in both steps, ~53% a mixture of walking and running, and ~15% walking in both steps. The variation observed between individuals is random (Table 1).

Pigeons moving on the treadmill did not exhibit aerial phases. At speeds higher than 0.4 ms^−1^, pigeons still showed in 36% of the steps %congruity values lower than 50%. %. When comparing one step with the following step, on average ~31% of them exhibited running mechanics in both steps, ~53% a mixture of walking and running, and ~16% walking in both steps. Individual differences are significant due to one individual (200), which used relatively more walking and less grounded running during trials (Table 1). The variation observed among the other three individuals is random.

We collected data from quails locomoting at speeds ranging from 0.15 ms^−1^ to 0.85 ms^−1^. Most of the collected data was at speeds lower than 0.6 m s^−1^. Similar to the pigeons, the quail did not exhibit aerial phases within that speed range. At speeds higher than 0.4 ms^−1^, quail exhibited only in 14% of the steps %congruity values lower than 50%. When comparing one step with its following one, ~29% of them exhibited running mechanics in both steps, ~55% a mixture of walking and running, and ~16% walking in both steps. The variation observed between individuals is only due to chance (Table 1).

### Simulations

**Figure 3:**
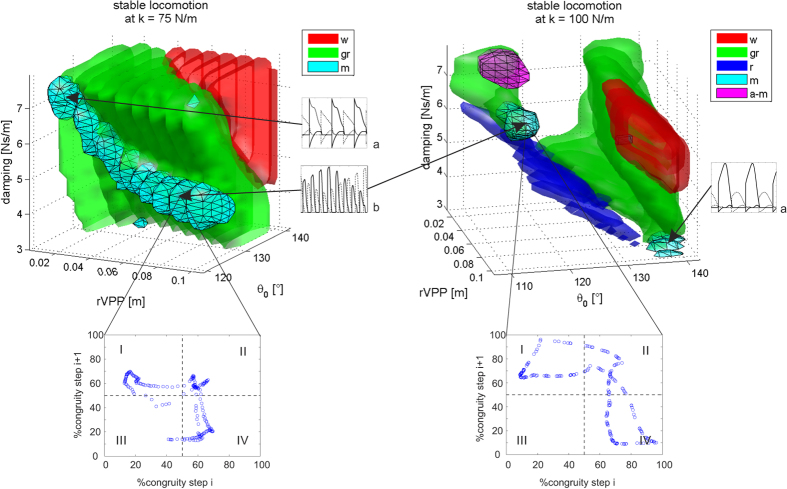
Fields of stable locomotion for walking-like (left) and bouncing-like (right) parameters (see methods). Grounded running (gr), running (r), walking (w), mixed (m), and apparently mixed (a-m). Mixed gaits are: (**a**) preferred-leg mixed gait, a combination of one step walking and one step grounded running, or (**b**) alternating mixed gait, the legs alternate gradually between walking and running mechanics(i.e. while one leg changes from walking to grounded running, the contralateral leg changes in the opposite way). Apparently mixed gaits converge to grounded running after ca. 1000 steps. Subplots display congruity changes between one step and the next for two examples of alternating mixed gaits. For every simulation, both legs have same leg parameters, such as length at touch-down *l*_*0*_, stiffness *k*, and damping *c*.

We simulated quail locomotion using the minimalistic PVPP model and parameters introduced in^21^. Results presented below are stable simulations. Stable walking, grounded running, and running were already presented in^21^. Here, we add results for mixed gaits. In simulations, we found two different types of mixed gaits, and an apparently mixed gait. In the preferred–leg mixed gait, one leg exclusively exhibits walking mechanics and the other only running mechanics (Fig. 3). In alternating mixed gait, both legs gradually shift, out of phase, between walking and running mechanics. An apparently mixed gait converges very slowly to grounded running, thus after small perturbations, the gait seems to be mixed (even after 100 simulated steps, Fig. 3, k = 100 Nm^−1^, magenta).

#### Mixed gaits in simulations using walking-like parameters

Using parameters obtained from walking quail such as effective leg stiffness and damping, average speed of locomotion (see methods), simulations yielded mixed gaits at speeds ranging from 0.17 ms^−1^ to 0.65 ms^−1^.

The mixed gait domain is a long, inclined volume that comprises almost the whole range of VPP heights (Fig. 3 left, cyan volume with black mesh). For lower VPP height, damping values are the highest and decrease with increasing VPP height. The domain also stretched from mean trunk angles between 120° and 135°. Interestingly, the mixed gaits domain is located far from parameters that induce the model to exhibit walking. The latter were observed at a more prone trunk position. Mixed gaits made up 5% of the total solutions.

#### Mixed gaits in simulations using bouncing-like parameter**s**

Using parameters obtained from bouncing-like quail gaits (see methods and table 1), mixed gaits existed in five small separated volumes (Fig. 3 right, cyan volumes with black mesh). The largest one is located at damping values exceeding 6.5 Nsm^−1^ with relatively shallow trunk angles between 112° and 120° and VPP heights up to 0.03 m. Again, mixed gait domains are located far from parameters which induce the model to exhibit walking. Using bouncing-like parameters, mixed gaits made up only 2% of the total solutions.

**Figure 4:**
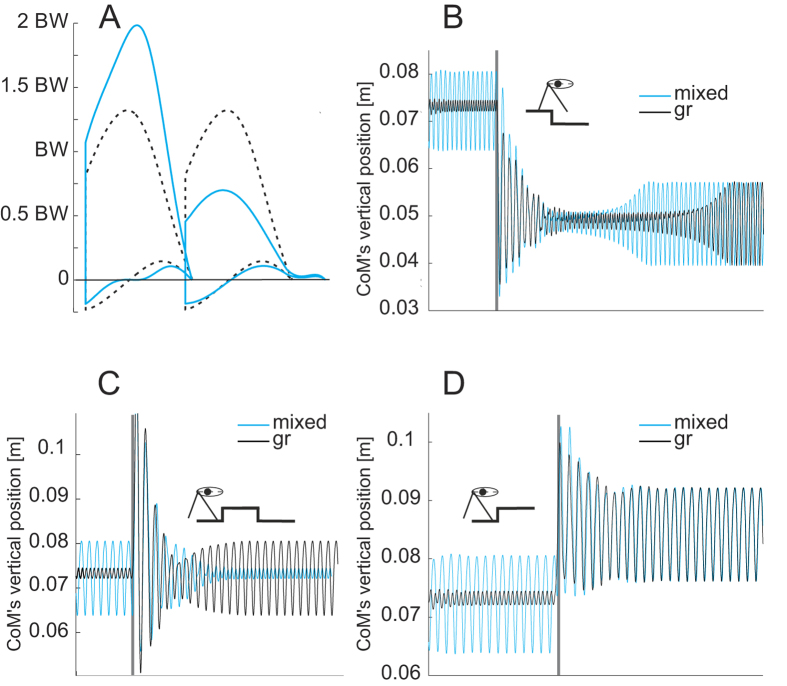
Switching between preferred-leg mixed gait and grounded running. In the overlapping zones of Fig. 3, two limit cycles exist for the same model parameters. (**A**) Model ground reaction forces (grounded running dashed lines, mixed gait solid line). (**B**) Step down simulation, grounded running (black) and preferred-leg mixed gait (cyan). In the simulations presented here model parameters are equal. During unperturbed locomotion (**B**, before vertical grey line), just the leg compression decides whether the gait converges to grounded running (y_0_ = 0.088) or to a mixed one (y_0_ = 0.074). The %congruity values after 50 steps are: grounded running (leg 1 = leg2 = 89); mixed (leg1 (A, red solid line) = 31.6, leg2 (**A**, black solid line) = 84.2). After step up (**D**) or step down (**B**) perturbation, both gaits converge to the same preferred-leg mixed gait; however, if the gait is mixed before the step-down perturbation, after the perturbation it converges much faster to the fixed point. After step-up-step-down perturbation (C, 20% leg length), grounded running converges to preferred-leg mixed and preferred-leg mixed to grounded running. Parameters: *Ψ*_0_ = 14_0_°, *r*_*VPP*_ = 0.07 m, *k* = 100 Nm^−1^, and *c* = 2.9 Nsm^−1^, ϕ_0_ = 5_0_.

Independent of parameters being walking- or bouncing-like, the mixed gait produces an imbalance in the GRFs. In Fig. 4A we present a comparison between the GRF obtained for grounded running and for preferred-leg mixed gait with identical model parameters though differing leg compression. During this mixed gait, the peak of the GRF measured in the grounded running leg increased by 54% while maximal value of the vertical GRF in the vaulting leg decreased by the same value. Both gaits oscillate about similar system energy (SE_gr_ ~ 0.246 J; SE_m_ ~ 0.248 J); however the dimensionless specific cost of transport is about 20% higher for mixed gait (CoT_gr_ = 0.211, CoT_m_ = 0.254).

## Discussion

We asked whether small birds predominantly use vaulting mechanics at lower speeds, and bouncing mechanics at the higher speeds. Alternatively, small avian locomotion is less discrete and permit the existence of mixed gaits such as those in which one step with walking mechanics is followed by one step running mechanics or *vice versa* at relative constant speed. To test this with reasonable degree of generality, we conducted an automated large-scale study on CoM movements in a limited number of relatively small, phylogenetically and morphologically diverse species of neognath birds (quail, oystercatcher, northern lapwing, pigeon, and avocet), using a recently published methodology^24^.To complement the experimental data, we also modelled gaits at the transitions between walking, grounded running, and running based on parameters derived from live quail (cf.^21^). Still, further studies including data from paleognath birds, i.e., tinamous and ratitites, will be necessary to test the broader generality of the results presented here.

Our experimental results show no clear correlation between %congruity and speed. This suggests that at least the birds studied do not strictly prefer walking mechanics at lower speeds or running mechanics at higher speeds. Moreover, our results clearly display that the birds in our study engage mixed gaits more often than walking and, surprisingly, maybe more/as often than/as grounded running (Table 1, Fig. 2). In general, our statistics show that a bird displaying a walking step on the treadmill only had a chance of ca. 15% to repeat walking in the subsequent step. In contrast, if it exhibits a bouncing step, the probability to exhibit again a bouncing step rises to ca. 40%. Thus, for large sequences, as shown in Fig. 2, there is a high chance to see combinations of different gaits. Accordingly, we found only in shorter video sequences (5 to 10 steps) singular gaits such as walking, grounded running or mixed gaits (see Fig. S2).

Recent studies have already shown that some small to medium sized avian species such as lapwings or tinamous do not always exhibit walking mechanics at lower speeds^7^,^10^,^11^. Other studies have shown that grounded running may be the most frequently used gait in avian locomotion^11^,^15^,^18^. These experimental observations might not be entirely explained by a more compliant leg in small birds. It has been shown that in mammals, leg stiffness scales as the two-third power of body mass^25^,^26^, regardless of whether this is quantified using a virtual leg defined as the length between the CoM and foot contact point or using the effective leg. In other words, leg stiffness is independent of body mass when normalized by body mass and leg-length^27^. Thus, more probably, birds engage running-like or mixed gait mechanics often because of their pronograde trunk orientation. Andrada and colleagues recently showed through numerical simulations with the PVPP model that most of the stable solutions obtained using quail parameters are indeed grounded running^21^. These results and those presented here add a functional criterion, namely the need for stability, to the frequently observed occurrence of grounded running and, as demonstrated in the current study, mixed gaits.

A clear separation between vaulting and bouncing mechanics can be useful to describe human locomotion. However, birds show a greater dispersion in the %congruity values. One explanation for these findings may lie in the pronograde trunk position in birds. Together, trunk, neck, and head make more than 80% of the total mass of an individual, and have a relative motion with respect to the leg (the trunk oscillates about the hip) so that leg compression and releases do not necessary oscillate in phase with trunk rotation^10^,^20^,^28^,^29^. Such a phase shift may vary based on the relation between the inertia of the trunk, the torque in the hip joint, and viscoelastic properties in the leg. It follows, that different anatomies may favor diverse relationships between potential and kinetic energy. Taking a closer look at the scatter plots, hints of species-related differences become evident. Northern lapwings and especially avocets, move mostly at %congruity values between 40 and 70 independently of speed, and choose bouncing gaits as often as mixed gaits if not more frequently. Such a locomotion may be distinctive for birds with relatively long legs and svelte bodies. The rather bulky pigeon and oystercatcher displayed a different pattern. %congruity values mostly indicate bouncing mechanics at lower speeds. Interestingly, as speed increased, the dispersion of the %congruity values increased, and even at higher speeds (see Fig. 1), they exhibited vaulting mechanics in ca. 40% of the steps. Quail are predominantly terrestrial birds; however, they did not show a clear trend towards vaulting or bouncing during locomotion on the treadmill. Quail, pigeon and oystercatcher seem to employ mixed gaits more frequently than bouncing gaits.

The scarcity of the red points in Fig. 1 indicate that running with aerial phases represent a rare event in our experiments. In our dataset, quail and pigeon did not use aerial phases. Here, the fact that they moved at dimensionless speeds (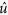) lower than 1 may explain the absence of aerial phases. Our quail subjects were mostly not cooperative to locomote at speeds higher than 0.7 m s^−1^. Aerial phases were nonexistent or rare in former quail treadmill and trackway studies^11^,^18^.

Our results suggest that mixed gaits are a ubiquitous feature in neognath avian locomotion. Therefore, it is reasonable to analyze numerically the existence of mixed gaits with input data of one species. The PVPP model incorporates three key features of bird locomotion: (i) the pronograde trunk, (ii) asymmetric compliant leg behavior along the leg axis, modelled as a parallel spring-damper system, and (iii) a fixed aperture angle as a leg alignment strategy. All three features were inferred from *in vivo* experiments involving quail (for more details see^21^). By simulating quail gaits using the minimalistic PVPP model in conjunction with parameters obtained through experiments with live quail, we were able to find different stable mixed-gaits: i) “preferred-leg mixed gait”, in which one step walking is followed by one step grounded running or *vice versa*, or ii) “alternating mixed gait”, in which a gradual shift from one gait mechanics into the other, occurring out of phase for each leg. These two types of mixed gaits emerged with both legs having same leg length (*l*_0_), stiffness (*k*), and damping (*c*) parameters. Interestingly, no matter whether leg parameters are vaulting or bouncing like, the parameter spaces belonging to mixed gaits are not located close to the boundaries between walking and grounded running, but rather at the boundaries between grounded running and running (Fig. 3). This should not be understood only in terms of speed, which is a state of the model. In fact, in contrast to^12^,^16^, our model found mixed gaits in a wide range of middle to higher speeds. In agreement with the model predictions, both mixed gaits can be observed in the experimental data. An example of preferred-leg mixed gait is shown in the ESM Fig. S2, while the example presented in Fig. 2 can be understood as an alternating mixed gait (cp. Fig. 3).

Our simulation findings may explain the remarkable dispersion in the %congruity values observed in our experimental data. Recall that mixed gaits parameters mostly overlap those of grounded running (Fig. 3). This means that in the overlapping zones, for the same combination of parameters, two limit cycles may exist (two stable periodic solutions). In those cases, simply changes in a state variable such as leg compression or perturbations in CoM height may induce grounded running to change to mixed gaits or *vice versa*. One example is presented in Fig. 4. There, both solutions exhibit similar system energy, but the management of the energy-flow among legs and trunk vary. In addition, the subplots in Fig. 3 reveal that if birds engage alternating mixed gaits, the same gait will be categorized either as mixed, grounded running, or even walking, depending only on the moment of the observation.

To what degree locomotion on normal ground vs. treadmills differs among avian species is a question that remains unresolved. Our results discussed here are not free from the issue of potential treadmill artifacts in the data (i.e. birds not moving normally due to the substrate moving beneath them). On the other hand, locomotion across tracks is frequently unsteady and limited to freely selected speed ranges and a few number of observed steps. Nevertheless, simulations support to a certain degree that mixed gaits are part of the dynamics of pronograde bipedal locomotion and not just a treadmill artifact.

However, our model predicts mixed gaits for only 5% of the solutions when using walking-like parameters and only 2% when using bouncing-like parameters, while the measured birds employ it in a large percentage of their strides. We think there are some possibilities to explain this paradox: a) birds might use very frequently a combination of leg parameters that are close to the attraction zone of mixed gaits, b) locomoting without visual flow might represent a disturbance to the animal enforcing such mixed gait patterns due to the relation between trunk motions and head bobbing^20^,^29^, or c) our simple 2D model, which has identical leg parameters and lacks of medio-lateral motions, fails in showing the complete solution space for mixed gaits. To investigate possibility ‘b,’ experiments with projected environments moving matched to the treadmill’s speed would be helpful.

Finally, the data presented here raises the question of the importance or advantages, for example in terms of energy or stability, that mixed gaits might offer robotics in transitioning between grounded running and running or in improving efficiency. At first view, mixed gaits seem to confer disadvantages in terms of visual cue (cf. discussion in^10^,^20^) and leg load, because of the larger CoM vertical amplitudes and GRF imbalance. In addition, our simulations indicate that mixed gaits are less efficient than grounded running at same system energies. The 20% higher requirement of energy obtained from our simulations poses new questions about trade-offs between metabolic optimality and stability that cannot be completely resolved in this study. However, if metabolic optimality was the only determining factor, one might expect that small birds would avoid mixed gaits completely. This expectation was clearly not supported in our experimental results. One way to mitigate the negative metabolic effects of mixed gaits might be to limit/reduce the %congruity imbalance between legs (see Supplementary Information Fig. S2). For small animals the terrain is almost always uneven^30^, and thus, for some leg settings, mixed gaits might emerge as the only self-stable way of movement.

## Methods

The main goals of the study were to determine how the congruity of CoM mechanics correlate with speed and if small birds really use mixed gaits. In light of this, we used available X-ray videos from the server of the Institut für Spezielle Zoologie und Evolutionsbiologie in Jena from a range of species across the avian phylogeny. By sampling a limited number of phylogenetically diverse (we sampled species from three clades: galliformes, columbiformes, and charadriiformes) and morphologically diverse (we included three species of shorebirds with highly differing limb segment proportions), we could reasonably discern with a limited sample of just a few species whether shift of %congruity and mixed gaits are a more ubiquitous feature of small avian terrestrial locomotion and not a peculiarity of isolated species. Note that our study included only neognath birds, therefore, similar studies on tinamous and ratities will be necessary for a higher level of generality. The methods reported below describe in general the experimental setup. For the purposes of this study, we reanalyzed previously recorded data.

Our large scale analysis covers 757 treadmill trials of five species (common quail: *Coturnix coturnix*, Eurasian oystercatcher: *Haematopus ostralegus*, northern lapwing: *Vanellus vanellus*, domestic pigeon: *Columba livia*, and pied avocet: *Recurvirostra avosetta*), totalizing 7749 steps and 1,267,320 image frames. Animals were obtained from local breeders and housed in spacious cages with access to food and water ad libitum at the Institut für Spezielle Zoologie und Evolutionsbiologie in Jena, Germany. The Committee for Animal Welfare of the State of Thuringia, Germany, approved the animal keeping and all experimental procedures (registry number 02-47/10). Animals were kept and all experiments were carried out in strict accordance with the approved guidelines.

The treadmill was covered with a tunnel made of Plexiglas so that the animals were not able to fly away from the treadmill. When the treadmill was started the animals usually started to walk on their own. While walking and running on the treadmill at different speeds, high-speed X-ray videos were recorded from the lateral and ventral projection (fully digital X-ray device Neurostar, Siemens AG, Erlangen, Germany). Additionally, two standard light cameras were synchronized with the X-ray camera and filmed from a fronto-lateral and a frontal perspective. All recordings were obtained at a rate of either 0.5 kHz or 1 kHz. Each frame was taken as a digital image with a resolution of 1,536 × 1,024 pixels (X-ray camera) and 512 × 512 pixels (standard light cameras), respectively. The X-ray source operated at 40 kV and 95 mA. Prior to analysis all X-ray recordings were undistorted using a freely available MATLAB (The MathWorks Inc., Natick, MA, USA) routine (www.xromm.org) provided by Brown University (Providence, USA).

We are aware that locomotion is a 3D phenomenon and that birds do not operate limbs only parasagittally^29^,^31^,^32^. However, for the determination of a gait, it is sufficient to use CoM oscillations in the sagittal plane (e.g.^2^,^3^,^16^,^18^,^33^,^34^). Thus, for the automatic estimation of CoM position and %congruity we only used the lateral projection of the available X-ray videos.

### Removing background and automated CoM estimation

The method published by Haase and colleagues^24^ is able to approximate the position of the CoM without any kind of user interaction, solely based on the automatic analysis of the images of a given X-ray sequence. The main idea of this approach is to relate each pixel of an image to the mass of matter it represents. The theoretical basis for this process is the Beer–Lambert law^35^,^36^, which describes the absorption of electromagnetic waves traversing an arbitrary material. To resolve ambiguities arising from the fact that only one X-ray view is available (cf.^36^,^37^), this method assumes volumic mass and X-ray absorption coefficients to be identical for all materials, but relative errors caused by this approximation are small for materials encountered in X-ray animal analysis (namely water, fat, tissue, and bone). As the CoM calculation is solely based on image gray values, it is crucial to remove all background information from the images. Based on the Beer–Lambert law, a known background component (e.g. obtained by recording an empty sequence) can be removed from an image via a pixel-wise division by the background image. However, in many cases such as for previously recorded datasets, the background is not known and thus has to be estimated from the image sequence itself. Because this is an ill-posed problem (i.e. an infinite set of solutions exists), we regularize the estimation to maximize the information explained by the background. The regularized problem has a unique solution which can efficiently be computed as the pixel-wise maximum overall images of a sequence. Artifacts in the background estimation may appear when the animal remains relatively static over the course of an entire sequence, but can easily be corrected using inpainting techniques (e.g.^38^). For more detailed derivation and discussion of the algorithm please refer to^24^. An implementation of the algorithm can be found for Matlab and C + + free for use under http://www.inf-cv.uni-jena.de/locomotion.

### Automated detection of foot contacts, aerial phases, and visibility of bird

**Figure 5:**
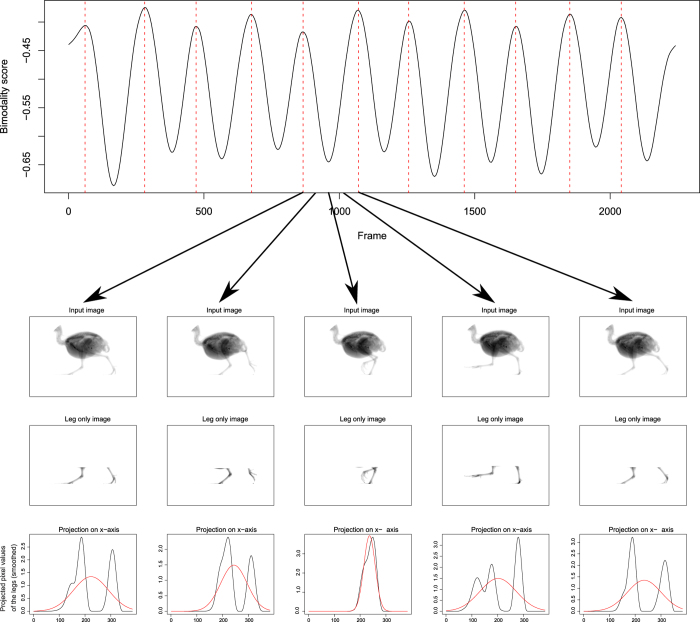
Touch-down detection for an exemplary quail sequence. The plot in the upper part of the figure shows the computed bimodality scores for each frame of the sequence. Touch-down is detected at local maxima of the score (marked by vertical dotted lines). For each frame, the bimodality score is assessed by (1) projecting the pixel values of the legs onto the x-axis, and (2) using the histogram intersection distance to compare the resulting distribution to a Gaussian distribution of same mean and variance (red curves). Examples of this process are shown in the lower part of the figure for five frames between two touch-down events. The leg-only images are computed automatically based on a projection of the pixel values onto the y-axis.

Touch-down detection: we found that when the pixel values of the leg of every frame are projected onto the x-axis, the obtained 1D-curve display at TD a maximal bimodality. We measured the bimodality value of the 1D-curve by comparing it to a normal distribution of same mean and variance using histogram intersection distance. Frames in which a TD event occurs are then found by identifying all local maxima of the bimodality score over all frames (Fig. 5).

Aerial phase: for each frame of a trial, we computed the minimum distance between the lowest point of the legs and the bottom of the image. Afterwards, a threshold was used to classify each frame as “aerial phase” or “stance phase” based on its leg height. For each sequence, the threshold was estimated automatically in such a way that only frames in which the legs are substantially higher than the median leg height are classified as “aerial phase” (see Supplementary Information, Fig. S1).

Visibility: to account for cases in which the birds left the field of view, for every frame of each trial we computed the number of pixels in which the bird is visible (‘bird-pixels’). % of congruity was computed, only when the measured number of bird-pixel during all frames of a stance phase was higher than 80% of the maximal computed number of bird-pixels of the complete trial.

The algorithms described in this section were implemented in the programming language R and are freely available to download and use from http://dx.doi.org/10.6084/m9.figshare.1471629.

### Percentage of congruity

To differentiate vaulting from bouncing gaits, we used %congruity^19^. %congruity is the percentage of overall frames making up a step which feature frame-to-frame changes in *E*_*p*_ and *E*_*k*_ of equal sign. Note that %congruity does not consider the magnitudes of mechanical energies, and therefore cannot account for the amount of energy conversion or recovery. On the other hand, this method permits the analysis of CoM dynamics without the need of calibration of the X-ray videos. Ideally, %congruity would be 100% in a perfect bouncing (running) trial and 0% in an ideal vaulting (walking) trial. *E*_*p*_ was calculated as


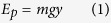


where *m* is mass, *g* is gravitational acceleration, and *y* is the vertical oscillation of the CoM. *E*_*k*_ was calculated as


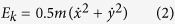


where 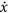 and 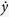 are the horizontal and vertical velocities, respectively. Note that mechanical energies are not in SI units.

For the sake of simplicity, we defined a step, as the period between the touch-down of a leg and the touch-down of the contralateral leg. For computing %congruity we did not include the first and last steps of each trial, because they are usually incomplete. Then, to avoid erroneous %congruity calculation due to stumbles or speed changes, we used only steps of a trial which exhibited similar contact times. To accomplish that, we computed the median of the contact periods of the steps of a trial (in frames). Only contact periods within ±10% of the median were used for further calculations. Further, we discarded steps if the step-to-step horizontal variation of the CoM was larger than 20 pixels (approx. 5 mm). Then, we used an elliptic high-pass filter to reduce negative effect of drift (2 Hz cut-off frequency) in the computation of congruity. Finally, we low pass filtered at 100 Hz both coordinates of the CoM.

For selected trials (one walking and one bouncing for each individual), we computed the relative position of the CoM related to the pelvis and the effective leg length. Effective leg length was computed in SI units. For that purpose, we digitized landmarks corresponding to the hip, distal part of the tarsometatarsus and tip of the middle toe from both lateral and ventral X-ray views using Winanalyse (Mikromac, Germany). A calibration object into which metal beads were inserted at known distances was also recorded in both projections at the end of each recording day. Direct linear transformation (DLT) was performed in Winanalyse to 3D calibrate the recorded space. Effective leg length was then computed in Matlab from the sagittal projection of the 3D-coordinates.

For appropriate comparisons of bipedal locomotion among the five species, we calculated the dimensionless speed (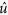), defined as


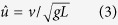


where *v* is the treadmill speed, *g* is the gravitational acceleration, and *L* is the effective leg length defined as the mean distance between the hip and the tarsometatarso-phalangeal joints during the stance phase.

### Statistics

To analyze whether the variation of the individual gait-preference observed among every species is due to chance we used the Chi-square test (PASW Statistics for Windows, Version 18.0. Chicago: SPSS Inc.; asymptotic method for expected frequencies above 5, exact method for frequencies below 5).

### Numerical model

**Figure 6:**
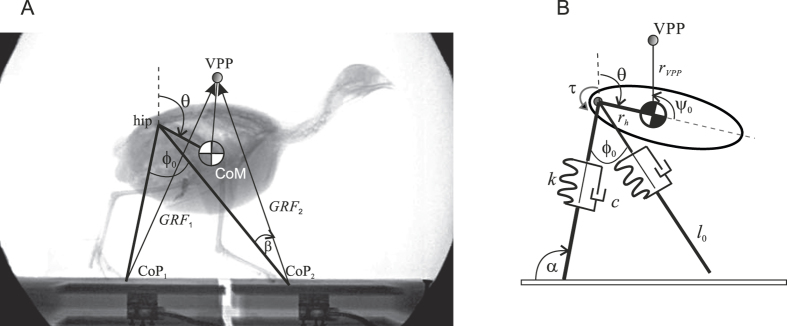
Bird and PVPP model. To obtain the necessary parameters for the PVPP model, synchronous X-ray videography and force measurements data from^21^ were used. (**A**) Lateral X-ray projection of a quail during stepping on two forceplates. Schematic drawing superimposed onto X-ray still image depicting experimental data analyzed for developing the model VPP, GRFs, effective legs (segments hip-CoP1, hip-CoP2), aperture angle between effective legs at touch-down ϕ_0_, trunk angle θ, angle between effective leg, and GRF β. (**B**) Minimalistic quail model using a VPP for postural control, and asymmetric leg behavior modeled as parallel spring and damper. τ hip torque, ψ_0_ angle between trunk and VPP, α angle between ground and effective leg, *k* leg stiffness, *c* leg damping, *l*_0_ rest length at touchdown, *r*_*h*_ distance hip-CoM, *r*_*VPP*_ distance CoM-VPP. Parameters *k*, *c*, *l*_0_ were optimized to best fit the measured force-effective leg length relationship during stance in the quail (see text and^21^ for further explanations).

The sagittal plane PVPP model^21^ consists of a rigid body of mass *m* with a moment of inertia *J* connected at the hip to two massless legs modeled as parallel spring-damper elements (Fig. 6B). The trunk can pivot freely about the hip axis. The CoM of the model is located at a distance *r*_*h*_ from the hip at an inclination *θ* from the vertical (Fig. 6B). The location of the VPP is given by the distance *r*_*VPP*_ from the CoM and the inclination 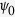 from the body axis (Fig. 6B). The equations of motion are:


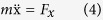



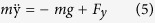



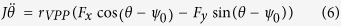


where *F*_*x*_ and *F*_*y*_ are respectively the sum of the horizontal and vertical components of the GRF of the legs, *g* is gravitational acceleration, 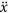, 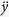, and 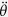 are the horizontal, vertical, and rotational CoM accelerations, respectively. The GRF is calculated to point towards the given VPP.

We use the same two gait categories of initial parameters used by Andrada and colleagues (2014), which is based on the parameters obtained from vaulting and bouncing gaits of live quail: (i) walking-like, with *k* = 75 Nm^−1^, ϕ_0_ = 45°, and horizontal initial velocity *v*_*x*0_ = 0.4 ms^−1^, and (ii) bouncing-like with *k* = 100 Nm^−1^, ϕ_0_ = 50°, and *v*_*x*0_ = 0.6 ms^−1^. Just as in the experiments with living animals, we relied on %congruity to discriminate vaulting from bouncing, and then distinguished running from grounded running by checking for aerial phases (i.e., when no leg has contact to the ground). A rigorous analysis of stability was not the aim of this paper. Also following our previous work^21^, we defined stability as the ability to cope with undetected perturbations of the ground level. Finally to compare efficiency between gaits, we used the dimensionless specific cost of transport, CoT = energy used/(weight x distance traveled) (7) (see ESM for more explanations on stability and CoT).

## Additional Information

**How to cite this article**: Andrada, E. *et al.* Mixed gaits in small avian terrestrial locomotion. *Sci. Rep.* **5**, 13636; doi: 10.1038/srep13636 (2015).

## Supplementary Material

Supplementary Video 1

Supplementary Video 2

Supplementary Information

## Figures and Tables

**Table 1:**
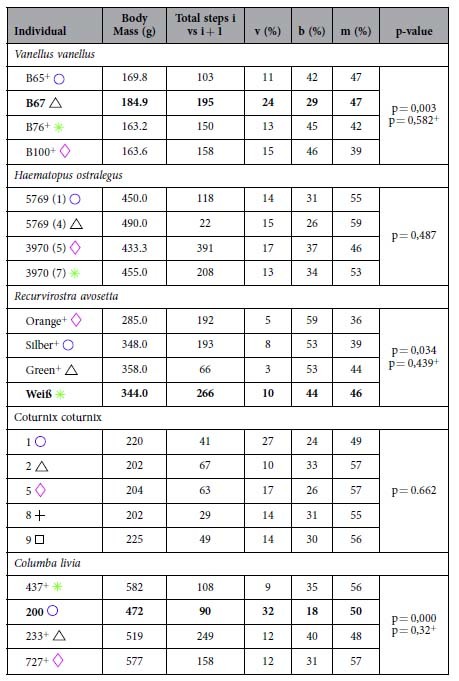
Individual data.

Body mass for individual study animals, total number of steps analyzed per individual, percentage of occurrence of vaulting mechanics (v), bouncing mechanics (b), and mixed gaits (m), and p-value chi^2 test. For pigeon, avocets, and northern lapwings is the p-value computed significant due to the variation of one individual (bold). The variation of the other 3 individuals (marked with^+^) among these species is due to chance (see p^+^).

## References

[b1] Hildebrand M. Symmetrical gaits of horses. Science 150, 701–708 (1965). 5844074 10.1126/science.150.3697.701

[b2] Cavagna G. A., Heglund N. C. & Taylor C. R. Mechanical work in terrestrial locomotion: two basic mechanisms for minimizing energy expenditure. Am J Physiol 233, R243–261 (1977). 411381 10.1152/ajpregu.1977.233.5.R243

[b3] Cavagna G. A., Thys H. & Zamboni A. The sources of external work in level walking and running. J Physiol 262, 639–657 (1976). 1011078 10.1113/jphysiol.1976.sp011613 PMC1307665

[b4] Biknevicius A. R. & Reilly S. M. Correlation of symmetrical gaits and whole body mechanics: debunking myths in locomotor biodynamics. J Exp Zool Part A: Comparative Experimental Biology 305, 923–934 (2006). 10.1002/jez.a.332 17029269

[b5] Blickhan R. The spring-mass model for running and hopping. J Biomech 22, 1217–1227, 10.1016/0021-9290(89)90224-8 (1989). 2625422

[b6] Gatesy S. M. & Biewener A. A. Bipedal locomotion: effects of speed, size and limb posture in birds and humans. J Zool 224, 127–147, 10.1111/j.1469-7998.1991.tb04794.x (1991).

[b7] Nyakatura J. A., Andrada E., Grimm N., Weise H. & Fischer M. S. Kinematics and Center of Mass Mechanics During Terrestrial Locomotion in Northern Lapwings (Vanellus vanellus, Charadriiformes). J Exp Zool Part A: Ecological Genetics and Physiology 317, 580–594, 10.1002/jez.1750 (2012). 22927254

[b8] Schmidt A. & Biknevicius A. R. Structured variability of steady-speed locomotion in rats. J Exp Biol 217, 1402–1406 (2014). 24436374 10.1242/jeb.092668

[b9] Abourachid A. & Renous S. Bipedal locomotion in ratites (Paleognatiform): examples of cursorial birds. Ibis 142, 538–549, 10.1111/j.1474-919X.2000.tb04455.x (2000).

[b10] Hancock J. A., Stevens N. A. & Biknevicius A. R. Whole-body mechanics and kinematics of terrestrial locomotion in the Elegant-crested Tinamou. Eudromia elegans. Ibis 149, 605–614 (2007).

[b11] Stoessel A. & Fischer M. S. Comparative intralimb coordination in avian bipedal locomotion. J Exp Biol 215, 4055–4069, 10.1242/jeb.070458 (2012). 22899525

[b12] Srinivasan M. & Ruina A. Computer optimization of a minimal biped model discovers walking and running. Nature 439, 72–75 (2006). 16155564 10.1038/nature04113

[b13] Geyer H., Seyfarth A. & Blickhan R. Compliant leg behaviour explains basic dynamics of walking and running. Proc. R. Soc. B 273, 2861–2867, 10.1098/rspb.2006.3637 (2006). PMC1664632 17015312

[b14] Shen Z. H. & Seipel J. E. A fundamental mechanism of legged locomotion with hip torque and leg damping. Bioinspiration & Biomimetics 7, 046010 (2012). 22989956 10.1088/1748-3182/7/4/046010

[b15] Andrada E., Rode C. & Blickhan R. Grounded running in quails: simulations indicate benefits of observed fixed aperture angle between legs before touch-down. J Theor Biol 335, 97–107 (2013). 23831138 10.1016/j.jtbi.2013.06.031

[b16] Usherwood J. R. Inverted pendular running: a novel gait predicted by computer optimization is found between walk and run in birds. Biol Lett 6, 765–768 (2010). 20484229 10.1098/rsbl.2010.0256 PMC3001358

[b17] Rubenson J., Heliams D. B., Lloyd D. G. & Fournier P. A. Gait selection in the ostrich: mechanical and metabolic characteristics of walking and running with and without an aerial phase. Proc. R. Soc. Lond. B 271, 1091–1099, 10.1098/rspb.2004.2702 (2004). PMC1691699 15293864

[b18] Andrada E., Nyakatura J. A., Bergmann F. & Blickhan R. Adjustments of global and local hindlimb properties during terrestrial locomotion of the common quail (Coturnix coturnix). J Exp Biol 216, 3906–3916 (2013). 23868846 10.1242/jeb.085399

[b19] Ahn A. N., Furrow E. & Biewener A. A. Walking and running in the red-legged running frog, Kassina maculata. J Exp Biol 207, 399–410, 10.1242/jeb.00761 (2004). 14691087

[b20] Nyakatura J. & Andrada E. On vision in birds: coordination of head-bobbing and gait stabilises vertical head position in quail. Front Zool 11, 27 (2014). 24666790 10.1186/1742-9994-11-27 PMC3987125

[b21] Andrada E., Rode C., Sutedja Y., Nyakatura J. A. & Blickhan R. Trunk orientation causes asymmetries in leg function in small bird terrestrial locomotion. Proc. R. Soc. B 281, 10.1098/rspb.2014.1405 (2014). PMC4240980 25377449

[b22] Roberts T. J., Marsh R. L., Weyand P. G. & Taylor C. R. Muscular force in running turkeys: the economy of minimizing work. Science 275, 1113–1115 (1997). 9027309 10.1126/science.275.5303.1113

[b23] Farris D. J. & Sawicki G. S. Human medial gastrocnemius force–velocity behavior shifts with locomotion speed and gait. Proc. Natl. Acad. Sci. U.S.A. 109, 977–982 (2012). 22219360 10.1073/pnas.1107972109 PMC3271879

[b24] Haase D., Andrada E., Nyakatura J. A., Kilbourne B. M. & Denzler J. Automated approximation of center of mass position in X-ray sequences of animal locomotion. J Biomech 46, 2082–2086 (2013). 23838281 10.1016/j.jbiomech.2013.06.009

[b25] Farley C. T., Glasheen J. & McMahon T. A. Running springs: speed and animal size. J Exp Biol 185, 71–86 (1993). 8294853 10.1242/jeb.185.1.71

[b26] Lee D. V., Isaacs M. R., Higgins T. E., Biewener A. A. & McGowan C. P. Scaling of the Spring in the Leg during Bouncing Gaits of Mammals. ICB 54, 1099–1108, 10.1093/icb/icu114 (2014). PMC4296203 25305189

[b27] Blickhan R. & Full R. J. Similarity in multilegged locomotion: Bouncing like a monopode. J Comp Physiol A 173, 509–517, 10.1007/bf00197760 (1993).

[b28] Hancock J. A., Stevens N. J. & Biknevicius A. R. Elegant-crested Tinamous Eudromia elegans do not synchronize head and leg movements during head-bobbing. Ibis 156, 198–208 (2014).

[b29] Abourachid A. *et al.* Bird terrestrial locomotion as revealed by 3D kinematics. Zoology 114, 360–368, 10.1016/j.zool.2011.07.002 (2011). 21982408

[b30] Fischer M. S. & Witte H. Legs evolved only at the end! Phil. Trans. R. Soc. A 365, 185–198 (2007). 17148056 10.1098/rsta.2006.1915

[b31] Rubenson J., Lloyd D. G., Heliams D. B., Besier T. F. & Fournier P. A. Adaptations for economical bipedal running: the effect of limb structure on three-dimensional joint mechanics. J. R. Soc. Interface 8, 740–755, 10.1098/rsif.2010.0466 (2010). 21030429 PMC3061092

[b32] Kambic R. E., Roberts T. J. & Gatesy S. M. Long-axis rotation: a missing degree of freedom in avian bipedal locomotion. J Exp Biol 217, 2770–2782, 10.1242/jeb.101428 (2014). 24855675

[b33] Heglund N. C., Cavagna G. A. & Taylor C. R. Energetics and mechanics of terrestrial locomotion. III. Energy changes of the centre of mass as a function of speed and body size in birds and mammals. J Exp Biol 97, 41–56 (1982). 7086349 10.1242/jeb.97.1.41

[b34] Daley M. A., Usherwood J. R., Felix G. & Biewener A. A. Running over rough terrain: guinea fowl maintain dynamic stability despite a large unexpected change in substrate height. J Exp Biol 209, 171–187, 10.1242/jeb.01986 (2006). 16354788

[b35] Kak A. C. & Slaney M. Principles of computerized tomographic imaging. (Society for Industrial and Applied Mathematics, 2001).

[b36] Buzug T. M. Computed tomography: from photon statistics to modern cone-beam CT. (Springer, 2008).

[b37] Tuy H. K. An inversion formula for cone-beam reconstruction. SIAM Journal on Applied Mathematics 43, 546–552 (1983).

[b38] Bertalmio M., Bertozzi A. L. & Sapiro G. Navier–Stokes, fluid dynamics,and image and video inpainting. In: Proceedings of the IEEE Conference on Computer Vision and Pattern Recognition (CVPR), Kauai, HI, USA. pp.355–362. IEEE, doi: 10.1109/cvpr.2001.990497 (2001, 12 8-14)

