# Supplementary material for: Mixed gaits in small avian terrestrial locomotion
Source: Sci Rep. 2015 Sep 3;5:13636. doi: 10.1038/srep13636 (PMC4558583; doi:10.1038/srep13636)
Supplement: Supplementary Information [file srep13636-s3.pdf]

Supplementary information:

Mixed gaits in avian terrestrial locomotion

Emanuel Andrada[1,3], Daniel Haase[2], Yefta Sutedja[1], John A. Nyakatura[3,4], Brandon Kilbourne[3,5], Joachim Denzler[2], Martin S. Fischer[3], Reinhard Blickhan[1].

Institutions:

[1]Science of Motion, Friedrich-Schiller University of Jena, Germany.

[2]Computer Vision Group, Friedrich-Schiller University of Jena, Germany

[3]Institut für Spezielle Zoologie und Evolutionsbiologie mit Phyletischem Museum, Friedrich-Schiller University of Jena, Germany.

[4] AG Morphologie und Formengeschichte, Bild Wissen Gestaltung: ein interdisziplinäres Labor, Institut für Biologie, Humboldt University Berlin, Germany.

[5]College for Life Sciences, Wissenschaftskolleg zu Berlin, Wallotstraße 19, 19143 Berlin, Germany.

S1 Material and methods (extended)

Table S1 Position of the center of mass (CoM), effective leg length (mean ± s.d.).

	Pos. CoM*	Effective leg length [m]
Quail	cc.= 0.48±0.03; vd.= -0.31±0.01	0.09±0.004
Avocet	cc.= 0.46±0.02; vd.= -0.23±0.04	0.179±0.005
N. Lapwing	cc.= 0.53±0.02; vd.= -0.08±0.03	0.13 ±0.006
Oystercatcher	cc.= 0.52±0.04; vd.= -0.14±0.02	0.151±0.011
Pigeon	cc.= 0.51±0.02; vd.= -0.15±0.02	0.117±0.002

*Pos. CoM: Relative position (from hip) of the bird`s center of mass is given as percentage of length between hip and caudalmost cervical vertebra. Values are positive in caudo-cranial (cc) and ventro-dorsal (vd) directions.

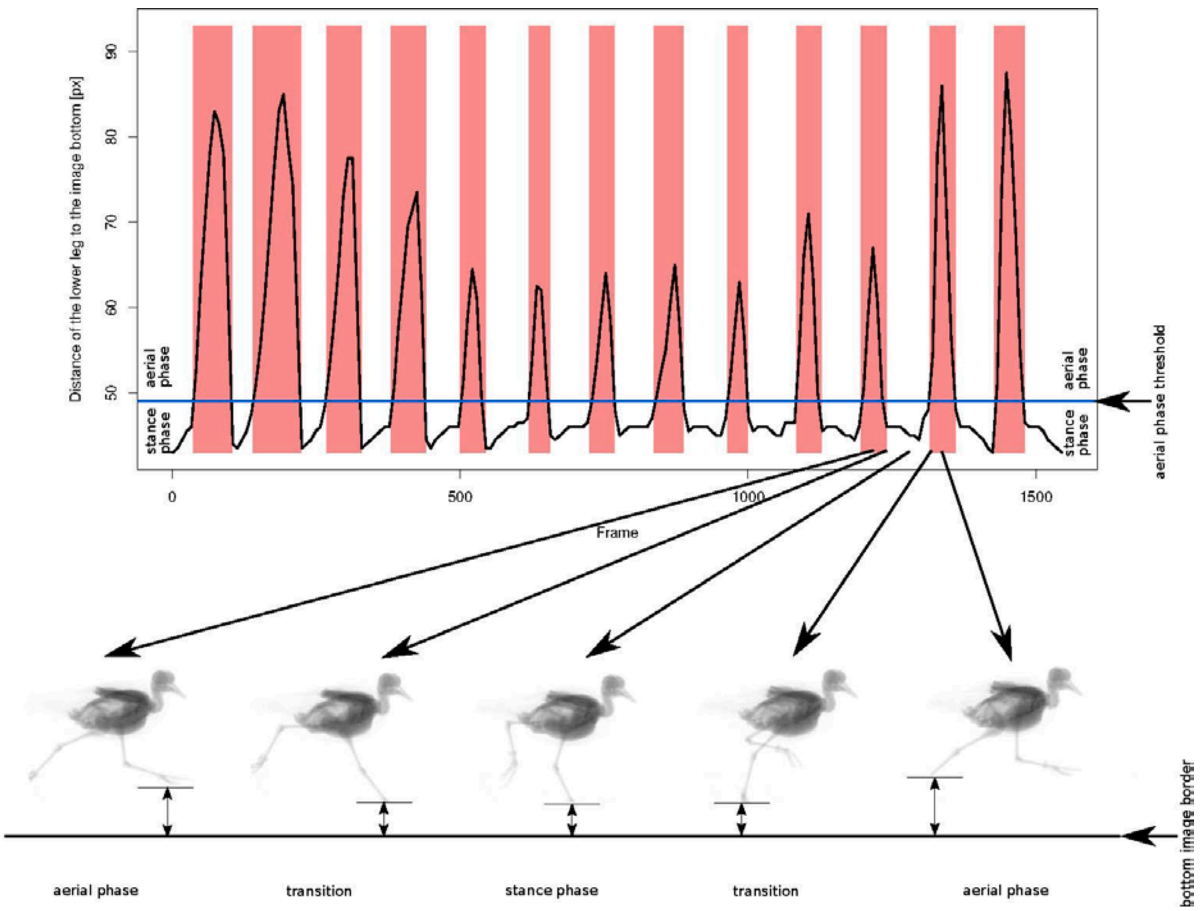

Figure S1: Aerial phase classification of an exemplary lapwing sequence. The plot in the upper part of the figure shows the minimum distance between the legs and the image bottom for each frame. The blue horizontal line indicates the automatically estimated threshold which is used to distinguish between "aerial phase" and "stance phase" for each frame. Frames which are classified as "aerial phase" are marked with a red background. Example images are shown in the lower part of the figure for five frames between two aerial phases. The code used for all automated detection tasks will be published free for use after the acceptance of the manuscript.

Simulations

Bipedal locomotion requires the regulation of hip torques to balance the trunk. In line with the tradition of keeping models as simple as possible to understand principles of locomotion [1], we extended the SLIP model by adding a trunk, controlling the hip torques such that the GRFs pointed to the VPP, and accounted for asymmetric leg function by using a damper in parallel to a spring-like leg. We call this model the Pronograde Virtual Pivot Point (PVPP) model[2].

PVPP model and geometry

Hip coordinates are given by

$$x_h = x - r_h \cdot sin\theta \qquad (1)$$

$$y_h = y + r_h \cdot cos\theta \qquad (2)$$

and touchdown occurs for

$$y_h = l_0 \cdot \sin(\alpha - \phi_0) \quad (3)$$

with x and y are the CoM coordinates in x- and y-axes, θ the trunk angle measured from vertical, α the angle between the posterior leg and the ground, and ϕ_0 the aperture angle (Fig. 2).

Finally, the position of the VPP is obtained as:

$$x_{vpp} = x + r_{vpp} \cdot \sin(\theta - \psi_0) \quad (4)$$

$$y_{vpp} = y + r_{vpp} \cdot \cos(\theta - \psi_0) \quad (5)$$

The GRF of each leg is required to point towards the VPP at all times. This is achieved by an additional force F_t acting at the tip of the leg perpendicular to the leg force F_a along the leg axis (Fig. 1). The GRF of each leg is the sum of F_a and F_t. The magnitude of F_a is defined by Eq. 1, while F_t is defined by $F_t = F_a \tan\beta$ (6), with β being the angle between leg and VPP (Fig. 2A). The hip torque is given by $M_h = F_t l$ (7).

Equations of motion

The equations of motion are:

$$m\ddot{x} = F_x \quad (8)$$

$$m\ddot{y} = -mg + F_y \quad (9)$$

$$J\ddot{\theta} = r_{VPP}\left(F_x \cos(\theta - \psi_0) - F_y \sin(\theta - \psi_0)\right) \quad (10)$$

Where F_x and F_y are the sum of the horizontal and vertical components of the GRF of the legs, g is gravitational acceleration, and $\ddot{x}$, $\ddot{y}$, and $\ddot{\theta}$ are the horizontal, vertical, and angular CoM accelerations, respectively.

Quail model parameters and parameter space

We constrained the scanned parameter space according to the experimental results: 1.5 $Nsm^{-1} \le c \le 8$ Nsm^{-1}, $\Delta c = 0.2$ Nsm^{-1}; $90° \le \psi_0 \le 140°$, $\Delta\psi_0 = 2°$; 0.01 m $\le r_{VPP} \le$ 0.1 m, $\Delta r_{VPP} = 0.005$ m; CoM initial height 0.07 m $\le y_0 \le$ 0.116 m, $\Delta y_0 = 0.002$ m. Note that during locomotion, the trunk angle θ oscillates about the ψ_0 value, i.e. $\theta_{mean} \approx \psi_0$. Initial speed for both categories is close to the mean values observed in the experiments (see [2]). Initial vertical speed is $v_{y0} = 0$ ms^{-1}, trunk angular speed is $\dot{\theta} = 0$ $rads^{-1}$, and the stance leg is oriented vertically. Initial trunk angle θ_0 is set to ψ_0.

Stability

A rigorous analysis of stability was not the aim of this paper. In accordance with literature [3], we defined stability as the ability to cope with even undetected perturbations. A common way of assessing gait stability is the steps-to-fall method [4]. We adopted a similar approach and analyzed whether the vertical amplitude of CoM movement was still decreasing in forward simulations after 100 completed steps (stable solutions converge asymptotically to the periodic oscillation). If this was the case, the simulation was deemed to be stable, though it might also be mathematical partially stable.

Cost of transport

We computed the dimensionless specific cost of transport, CoT= energy used/(weight x distance traveled) (11)

The energy used was inferred by computing the numerical time-integral of the hip power (area under the curve), which was obtained as

$$power = M_{hip}\dot{\theta}_{hip} \qquad (12)$$

Where M_{hip} is the hip torque and $\dot{\theta}_{hip}$ joint angular speed.

S2 Results

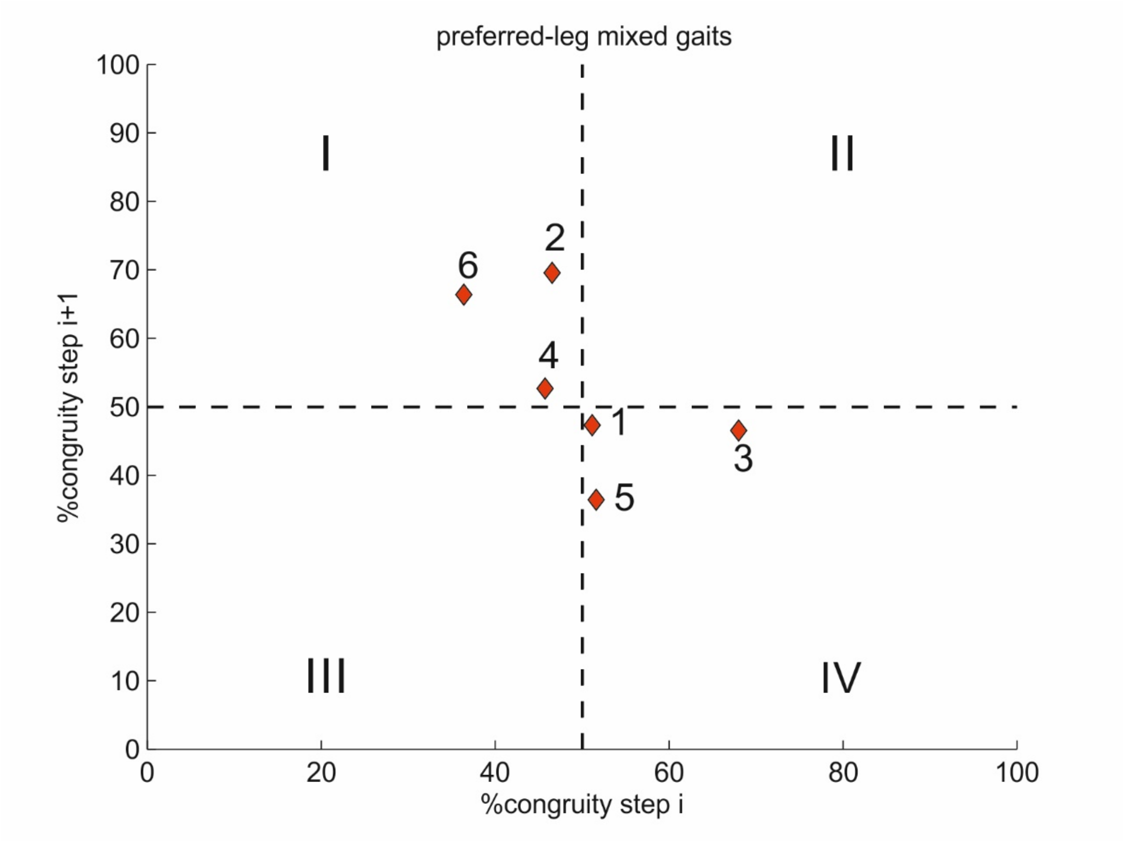

Fig S2. Values of %Congruity in step i vs. step i+1. Quadrant I and IV depict mixed gaits. In I %congruity shifts from walking values in step i to values representing running mechanics in step i+1. The inverse occurs in IV. II and III portray regular gaits of running and walking mechanics, respectively. Red diamonds represent a single trial from individual “Silber”. In this sequence of 7 steps, “Silber” uses only preferred-leg mixed gaits. Arabic numbers depict step sequence.

1 Full, R. J. & Koditschek, D. E. Templates and anchors: neuromechanical hypotheses of legged locomotion on land. *Journal of Experimental Biology* **202**, 3325-3332 (1999).

2 Andrada, E., Rode, C., Sutedja, Y., Nyakatura, J. A. & Blickhan, R. Trunk orientation causes asymmetries in leg function in small bird terrestrial locomotion. *Proceedings of the Royal Society B: Biological Sciences* **281**, doi:10.1098/rspb.2014.1405 (2014).

3 Blickhan, R. *et al.* Intelligence by mechanics. *Philos Transact A Math Phys Eng Sci* **365**, 199-220, doi:10.1098/rsta.2006.1911 (2007).

4 Seyfarth, A., Geyer, H., Gunther, M. & Blickhan, R. A movement criterion for running. *J Biomech* **35**, 649-655, doi:10.1016/S0021-9290(01)00245-7

(2002).
